# Intramedullary Nailing vs. Plate Fixation for Trochanteric Femoral Fractures: A Systematic Review and Meta-Analysis of Randomized Trials

**DOI:** 10.3390/jcm14155492

**Published:** 2025-08-04

**Authors:** Ümit Mert, Maher Ghandour, Moh’d Yazan Khasawneh, Filip Milicevic, Ahmad Al Zuabi, Klemens Horst, Frank Hildebrand, Bertil Bouillon, Mohamad Agha Mahmoud, Koroush Kabir

**Affiliations:** 1 Department of Orthopaedics and Trauma Surgery, Helios Wuppertal University Witten/Herdecke, 42283 Wuppertal, Germany; mghandourmd@gmail.com (M.G.); mohdyazan.khasawneh@helios-gesundheit.de (M.Y.K.); filip.milicevic@helios-gesundheit.de (F.M.); ahmad.alzuabi@hotmail.com (A.A.Z.); m.agha.mahmoud@gmail.com (M.A.M.); koroush.kabir@helios-gesundheit.de (K.K.); 2 Department of Orthopedics, Trauma and Reconstructive Surgery, RWTH Aachen University, 52056 Aachen, Germany; khorst@ukaachen.de (K.H.); fhildebrand@ukaachen.de (F.H.); 3 Department of Traumatology and Orthopedic Surgery, Cologne-Merheim Medical Center (CMMC), University Witten/Herdecke, 51109 Cologne, Germany; bouillonb@kliniken-koeln.de

**Keywords:** intramedullary fracture fixation, bone plates, femur, meta-analysis

## Abstract

**Background/Objectives**: Trochanteric femoral fractures pose significant surgical challenges, particularly in elderly patients. Intramedullary nailing (IMN) and plate fixation (PF) are the primary operative strategies, yet their comparative efficacy and safety remain debated. This meta-analysis synthesizes randomized controlled trials (RCTs) to evaluate clinical, functional, perioperative, and biomechanical outcomes of IMN versus PF specifically in trochanteric fractures. **Methods**: A systematic search of six databases was conducted up to 20 May 2024, to identify RCTs comparing IMN and PF in adult patients with trochanteric femoral fractures. Data extraction followed PRISMA guidelines, and outcomes were pooled using random-effects models. Subgroup analyses examined the influence of fracture stability, implant type, and patient age. Risk of bias was assessed using the Cochrane RoB 2.0 tool. **Results**: Fourteen RCTs (n = 4603 patients) were included. No significant differences were found in reoperation rates, union time, implant cut-out, or mortality. IMN was associated with significantly reduced operative time (MD = −5.18 min), fluoroscopy time (MD = −32.92 s), and perioperative blood loss (MD = −111.68 mL). It also had a lower risk of deep infection. Functional outcomes and anatomical results were comparable. Subgroup analyses revealed fracture stability and nail type significantly modified operative time, and compression screws were associated with higher reoperation rates than IMN. **Conclusions**: For trochanteric femoral fractures, IMN and PF yield comparable results for most clinical outcomes, with IMN offering some advantages in surgical efficiency and perioperative morbidity, though functional outcomes were comparable. Implant selection and fracture stability influence outcomes, supporting individualized surgical decision making.

## 1. Introduction

Trochanteric femoral fractures represent a clinically important subset of proximal femoral injuries, frequently occurring in elderly patients following low-energy trauma and posing substantial challenges in orthopedic practice [1,2]. Their management has evolved significantly, with intramedullary nailing and plate fixation being the two primary methods of internal stabilization [3,4]. While both approaches are widely accepted, the optimal fixation strategy remains a subject of ongoing debate, particularly given the complex biomechanics of the trochanteric region and the diversity in fracture morphology, patient profiles, and implant designs [5,6].

Importantly, while intertrochanteric, subtrochanteric, and trochanteric fractures are often grouped under the umbrella of proximal femoral fractures, they differ significantly in terms of anatomy, biomechanics, and treatment considerations [7]. Trochanteric fractures typically occur between the greater and lesser trochanters and are influenced by the pull of surrounding musculature, rendering them more rotationally unstable but more amenable to intramedullary fixation [7]. In contrast, subtrochanteric fractures extend distal to the lesser trochanter and are subject to high stress forces, often requiring alternative stabilization strategies [8]. Intertrochanteric fractures, though anatomically close, are usually more stable and less prone to complications associated with implant failure [9]. Given these differences, pooling these subtypes can lead to misleading conclusions; thus, our review focuses exclusively on trochanteric fractures to ensure biomechanical and clinical homogeneity.

Intramedullary nailing, a minimally invasive, load-sharing technique, has gained popularity due to its ability to preserve soft tissues, facilitate early mobilization, and offer biomechanical stability [3]. However, its use has been associated with complications such as technical insertion challenges, implant failure, and increased radiation exposure during fluoroscopic guidance [5]. Plate fixation—especially dynamic/sliding hip screws and locking plates—has also demonstrated favorable outcomes in stable trochanteric fractures due to its capacity for controlled compression and anatomic reduction [6,10]. Yet, this method typically requires larger surgical exposures, raising concerns about soft tissue disruption and infection risks [10].

Despite extensive clinical experience and numerous comparative studies, there remains no clear consensus on the superiority of either technique for trochanteric fractures. Prior meta-analyses have been limited by heterogeneity in study populations, inconsistent classification of fracture types, and inadequate subgroup analyses accounting for fracture stability and implant variability [11,12,13,14,15,16]. For instance, Parker et al. [17,18,19] reported favorable outcomes with nailing in trochanteric fractures, while others [20] emphasized the utility of sliding plates in subtrochanteric fractures—underscoring the need to disaggregate data by fracture subtype to avoid misleading generalizations.

Accordingly, the present systematic review and meta-analysis aims to address these limitations by focusing exclusively on randomized controlled trials (RCTs) comparing intramedullary nailing and plate fixation for trochanteric femoral fractures. By analyzing clinical, functional, biomechanical, and perioperative outcomes, and exploring potential effect modifiers such as fracture stability, implant type, and patient age, this study seeks to provide robust, fracture-specific evidence to support informed surgical decision making in this population.

## 2. Materials and Methods

### 2.1. Search Strategy

A systematic search of electronic databases (PubMed, Scopus, Web of Science, CENTRAL, ClinicalTrials.gov, and Google Scholar) was conducted on 20 May 2024, to identify RCTs comparing intramedullary nailing to plate fixation in trochanteric femoral fractures. The search strategy incorporated a combination of MeSH terms and free-text keywords including “trochanteric fracture”, “femur”, “hip”, “nail”, and “plate”. Google Scholar was limited to the first 200 records to maintain relevance [21]. Additional studies were identified through manual searches of reference lists, “similar articles” in PubMed, and prior systematic reviews [22]. Full details of the search strategy are provided in Supplementary Table S1. The study protocol was registered a priori on PROSPERO (CRD420251052676).

### 2.2. Eligibility Criteria

Studies were eligible if they met the following criteria:

- Design: Randomized controlled trials.
- Population: Adult patients with proximal femoral fractures involving the trochanteric region (i.e., fractures affecting the greater and/or lesser trochanter, with or without intertrochanteric extension). Studies focusing exclusively on subtrochanteric, femoral neck, or femoral shaft fractures were excluded.
- Interventions: Intramedullary nailing (e.g., proximal femoral nails, Ender nails, Gamma nails, etc.).
- Comparators: Plate fixation (e.g., sliding/dynamic hip screws, locking plates, compression plates).
- Outcomes: Reporting of at least one relevant clinical, functional, perioperative, or biomechanical outcome.

Given inconsistent use of the terms ‘intertrochanteric’, ‘pertrochanteric’, and ‘trochanteric’ in the literature, we relied on each study’s anatomical description and classification to determine inclusion eligibility, ensuring that only fractures centered in the trochanteric zone were included.

Studies were excluded if they involved other femoral fracture types (shaft, intertrochanteric, distal), used non-randomized designs, had overlapping datasets, or were review articles, abstracts, or biomechanical studies. No language or publication date restrictions were applied.

### 2.3. Data Extraction and Outcomes

Two independent reviewers extracted data from the included studies using a standardized data extraction form. The extracted information included study identifiers, sample size, patient demographics such as age and sex, fracture stability classification, the type of intramedullary nail or plate used, follow-up duration, and all reported outcome measures. Fracture stability was recorded as reported by each individual study. While some RCTs provided AO/OTA classifications (e.g., A1, A2, A3), others used narrative descriptions or did not classify stability. As no standardized or harmonized classification system was consistently applied across studies, we retained the original authors’ descriptions and reflected these in Table 1. Any discrepancies between reviewers were resolved by discussion or, if necessary, consultation with a third reviewer. The risk of bias for each included study was assessed using the Cochrane Risk of Bias 2.0 tool.

The primary outcomes evaluated in this meta-analysis were reoperation rate, time to fracture union, implant cut-out, complication rates (with specific focus on deep infection), and mortality. Secondary outcomes included operative time, fluoroscopy time, perioperative blood loss, the need for blood transfusion, and the length of hospital stay. Additionally, functional outcomes such as Harris hip scores and hip range of motion, as well as biomechanical parameters including femoral shortening, limb length discrepancy, and the occurrence of additional perioperative fractures or fissures, were analyzed. Where possible, subgroup analyses were performed to assess the influence of fracture stability, implant type, and patient age on the observed outcomes.

### 2.4. Statistical Analysis

Pooled meta-analyses were performed for outcomes reported in ≥2 studies using a random-effects model. Effect measures were odds ratios (ORs) for dichotomous outcomes and mean differences (MDs) for continuous outcomes, each with 95% confidence intervals (CI). Heterogeneity was assessed using the I^2^ statistic and Cochran’s Q test, with I^2^ >50% indicating moderate to high heterogeneity.

Subgroup analyses were conducted for fracture stability (stable vs. unstable), nail type, plate type, and patient age only when ≥3 studies reported outcome data for the respective subgroup. Subgroup or meta-regression analyses based on specific AO/OTA classes were not feasible due to inconsistent reporting and small numbers per subtype. Sensitivity analyses were performed by excluding high-risk-of-bias studies to evaluate the robustness of pooled estimates. Publication bias was assessed via funnel plots and Egger’s test where appropriate. All statistical analyses were conducted using STATA software (Version 18).

## 3. Results

### 3.1. Literature Review Results

**Figure 1:**
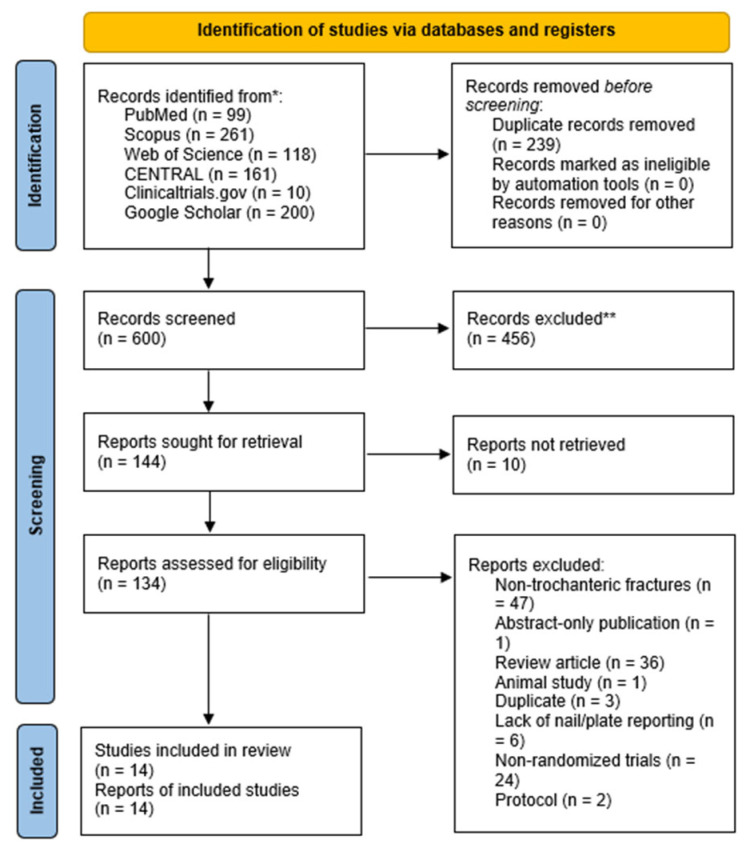
A PRISMA flow diagram showing the results of the literature search. * number of citations pre-duplicate removal through Endnote; ** number of citations post-duplicate removal.

The summary of the literature search and screening processes are provided in Figure 1. A total of 839 records were identified through database searches, including PubMed (n = 99), Scopus (n = 261), Web of Science (n = 118), CENTRAL (n = 161), ClinicalTrials.gov (n = 10), and Google Scholar (n = 200). After removing 239 duplicate records, 600 unique studies were screened based on titles and abstracts. Of these, 456 studies were excluded for irrelevance, leaving 144 reports for full-text review. During the full-text assessment, 134 reports were retrieved, while 10 reports could not be accessed. Following a detailed review, 73 studies were excluded for the following reasons: abstract-only publications (n = 1), non-trochanteric femoral fractures (n = 47), review articles (n = 36), animal studies (n = 1), duplicates not identified earlier (n = 3), lack of reporting on nailing or plating (n = 6), non-randomized study designs (n = 24), and protocols (n = 2). Ultimately, 14 RCTs met the inclusion criteria and were included in the qualitative and quantitative synthesis [18,19,23,24,25,26,27,28,29,30,31,32,33,34]. The search was updated on 27 May 2025, yielding no additional RCTs.

### 3.2. Baseline Characteristics of Included RCTs

The summary of the characteristics of included RCTs is provided in Table 1. Most evidence came from the United Kingdom (4 RCTs), with 4603 patients being examined (2270 in the intramedullary nailing group and 2333 in the plate fixation group). In terms of fracture stability, 4 trials included unstable fractures only, while the remaining included both stable and unstable without stratification. The follow-up period ranged from 1.5 months to 24 months, with a mean of 9.035 months. Nail- and plate-specific types can be found in Table 1.

### 3.3. Risk of Bias Assessment

**Figure 2:**
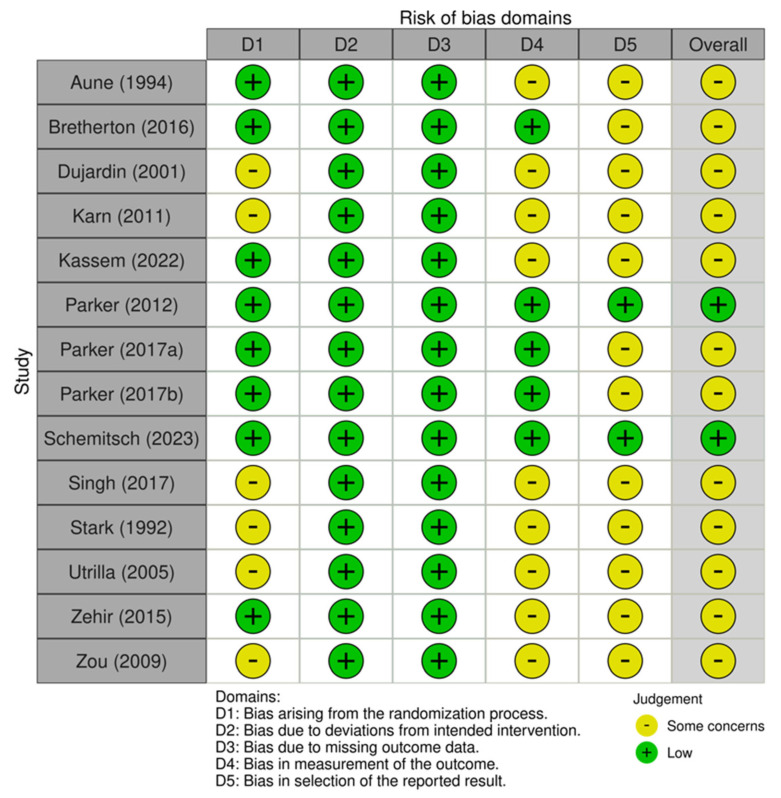
Illustration of the risk of bias of included randomized trials using the Cochrane’s revised tool (RoB-2) [18,19,24,25,26,27,28,29,30,31,32,33,34,35].

A summary of the risk of included trials is provided in Figure 2. Overall, only 2 RCTs had low risk, while the remaining 14 RCTs had some concerns. Specifically, blinding was reported only in 5 trials. In terms of trial registration, 12 trials (85.71%) were not registered on any international clinical trial platforms. Randomization method was adequate in 8 trials (57.14%), with 6 trials (42.86%) not providing information on randomization process.

### 3.4. Primary Outcomes

#### 3.4.1. Reoperation Rate

The meta-analysis of eight RCTs revealed no significant difference in reoperation risk between intramedullary nailing and plate fixation (OR = 1.15; 95% CI: 0.50, 2.63) (Figure S1). Moderate heterogeneity was present (I^2^ = 67.05%, *p* = 0.01), but sensitivity analysis showed no meaningful change in the effect estimate (Figure S2).

**Figure 3:**
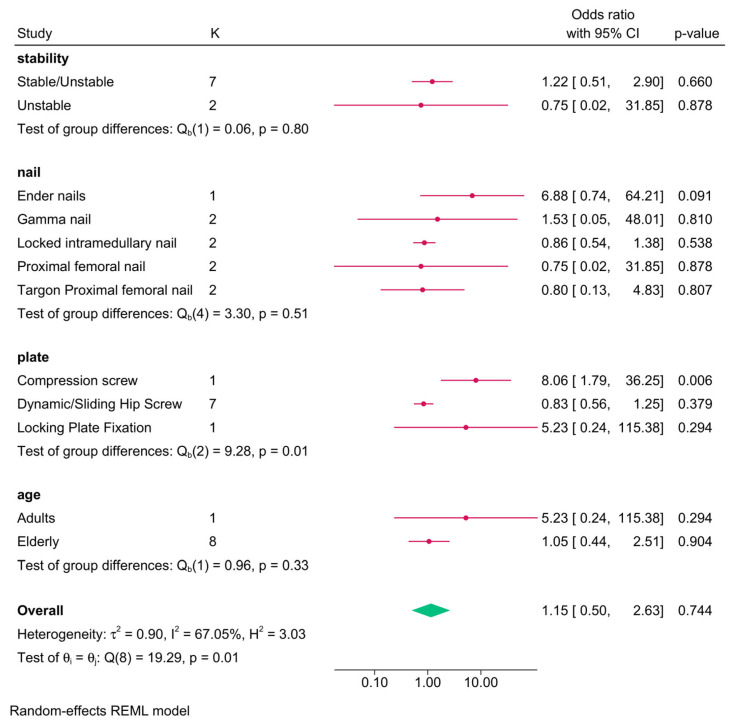
Forest plot showing the risk of reoperation in intramedullary nailing versus plate fixation in trochanteric fracture, stratified by nail/plate type, age, and fracture stability.

Subgroup analysis indicated that plate type significantly modified the effect (interaction *p* = 0.01). Compression screw was associated with a significantly higher reoperation risk compared to intramedullary nailing (1 RCT; OR = 8.06; 95% CI: 1.79, 36.25), while both dynamic/sliding hip screws and locking plates showed no significant difference. Other factors, such as fracture stability (*p* = 0.80), nail type (*p* = 0.51), and age (*p* = 0.33), did not significantly influence the outcome (Figure 3).

#### 3.4.2. Union Time (Months)

A pooled meta-analysis of three RCTs revealed no significant difference in union time (MD = −0.85 months; 95% CI: −2.79, 1.08) (Figure S3). Moderate heterogeneity was observed (I^2^ = 62.71%, *p* = 0.08), but it was not deemed significant.

#### 3.4.3. Implant Cut-Out

Seven RCTs assessed the risk of implant cut-out, revealing no significant difference between intramedullary nailing and plate fixation (OR = 0.70; 95% CI: 0.33, 1.52, I^2^ = 0%, *p* = 0.92) (Figure S4). Subgroup analyses did not identify any significant modifying effects.

#### 3.4.4. Complications and Mortality

Across eight RCTs, intramedullary nailing was associated with a significantly lower risk of deep infection (OR = 0.24; 95% CI: 0.08, 0.73, I^2^ = 0%). However, there were no differences in the rates of other complications, including avascular necrosis, deep vein thrombosis, hematoma, superficial infection, non-union, pulmonary embolism, pneumonia, or pressure ulcers (Table 2).

Mortality rates were also similar between the two groups (5 RCTs; OR = 1.08; 95% CI: 0.75, 1.54, I^2^ = 0%, *p* = 0.63).

### 3.5. Perioperative Outcomes

#### 3.5.1. Operative Time

**Figure 4:**
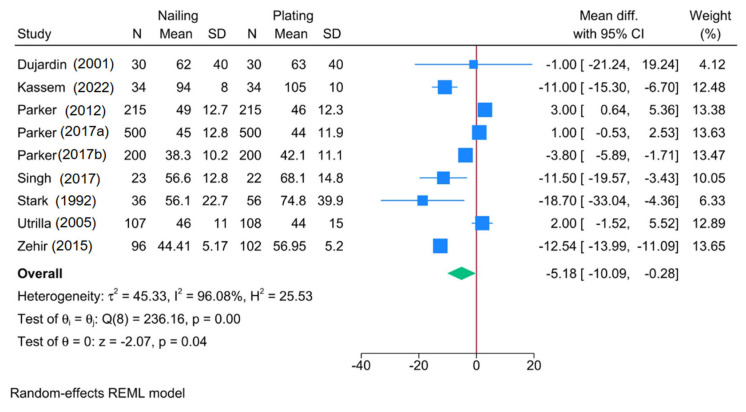
Forest plot showing the difference in operative time between intramedullary nailing versus plate fixation in trochanteric fracture [18,19,26,28,29,31,32,33,34].

Eight RCTs showed a significantly shorter operative time in the intramedullary nailing group (MD = −5.18 min; 95% CI: −10.09, −0.28) (Figure 4). Heterogeneity was substantial (I^2^ = 96.08%, *p* < 0.001). Sensitivity analysis revealed that the pooled effect became insignificant when certain RCTs were excluded (Figure S5).

**Figure 5:**
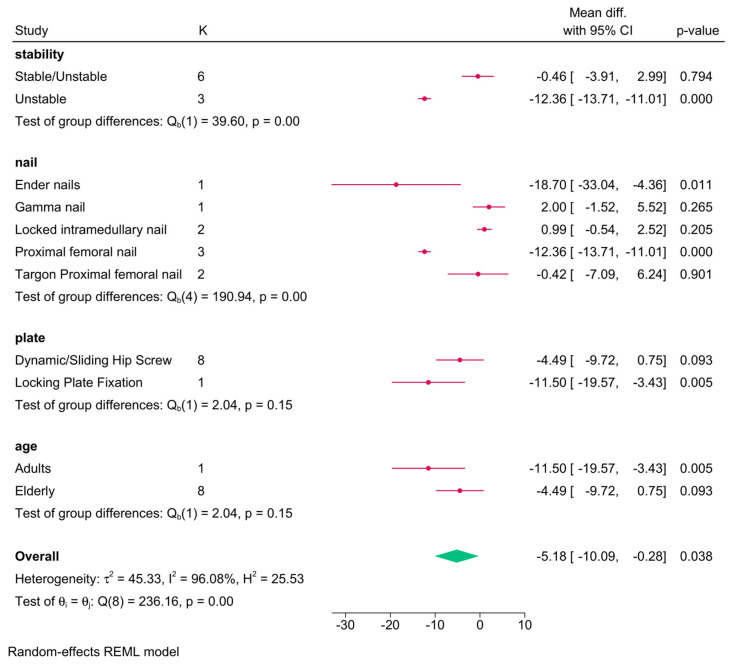
Forest plot showing the difference in operative time between intramedullary nailing versus plate fixation in trochanteric fracture, stratified by nail/plate type, age, and fracture stability.

Subgroup meta-analysis showed that fracture stability (*p* < 0.001) and nail type (*p* < 0.001) significantly influenced outcomes (Figure 5). Shorter operative time was observed with intramedullary nailing for unstable fractures (3 RCTs; MD = −12.36; 95% CI: −13.71, −11.01), but not for stable ones. Proximal femoral nails and Ender nails were associated with shorter operative times; Gamma, Targon, and locked nails were not. Plate type (*p* = 0.15) and age (*p* = 0.15) had no significant effects.

#### 3.5.2. Fluoroscopy Time

Two RCTs demonstrated significantly shorter fluoroscopy time in favor of intramedullary nailing (MD = −32.92 s; 95% CI: −36.15, −29.68; I^2^ = 0%).

#### 3.5.3. Perioperative Blood Loss

The meta-analysis of five RCTs showed significantly reduced perioperative blood loss with intramedullary nailing (MD = −111.68 mL; 95% CI: −180.40, −42.96) (Figure S6). Heterogeneity was high (I^2^ = 90.89%, *p* < 0.001), but sensitivity analysis revealed no change in effect. Subgroup analyses did not identify significant effect modifiers (all *p* > 0.4).

#### 3.5.4. Need for Blood Transfusion

Four RCTs showed no significant difference in transfusion rates between the two groups (OR = 0.91; 95% CI: 0.75, 1.09; I^2^ = 0%, *p* = 0.24) (Figure S7). However, most studies were based on elderly patients without clear fracture classification and used sliding hip screws as the control.

#### 3.5.5. Length of Hospital Stay

Five RCTs showed no significant difference in hospital stay (MD = −0.17 days; 95% CI: −2.24, 1.91; I^2^ = 60.18%, *p* < 0.001) (Figure S8). Sensitivity and subgroup analyses showed consistent findings (*p* > 0.05).

### 3.6. Functional and Anatomical Outcomes

#### 3.6.1. Harris Hip Score

The meta-analysis of two RCTs showed no significant difference between intramedullary nailing and plate fixation (MD = 2.38; 95% CI: −1.32, 6.07; I^2^ = 0%).

#### 3.6.2. Hip Range of Motion (ROM)

Two RCTs assessed hip ROM and found no significant difference (MD = 9.44; 95% CI: −5.88, 24.76; I^2^ = 88.97%, *p* < 0.001). Due to heterogeneity, sensitivity analysis was not feasible.

#### 3.6.3. Shortening

Two RCTs assessed femoral shortening and showed no significant difference (MD = −0.15 mm; 95% CI: −2.99, 2.69; I^2^ = 99.75%, *p* < 0.001). Sensitivity analysis was not feasible.

### 3.7. Implant-Related Complications

#### Additional Perioperative Fractures/Fissures

Eight RCTs showed no significant difference in the risk of additional perioperative fractures between groups (OR = 1.52; 95% CI: 0.60, 3.86; I^2^ = 0%, *p* = 0.57) (Figure S9). Subgroup analyses revealed no significant moderation by fracture stability (*p* = 0.99), implant type (nail *p* = 0.40, plate *p* = 0.30), or age (*p* = 0.30) (Figure S10).

## 4. Discussion

This systematic review and meta-analysis synthesized evidence from 14 randomized controlled trials comparing intramedullary nailing and plate fixation in trochanteric femoral fractures. Our findings demonstrated that both techniques offer comparable outcomes for key endpoints such as union time, reoperation risk, implant cut-out, and mortality. However, intramedullary nailing was associated with advantages in operative time, reduced deep infection rates, and lower perioperative blood loss, which may translate into clinically relevant benefits in selected patient populations.

The lack of significant differences in reoperation rates or union times aligns with earlier meta-analyses that failed to demonstrate consistent superiority of either technique when broadly applied across trochanteric fractures [17]. However, our subgroup analyses suggest that implant choice—particularly plate type—can substantially influence the risk of reoperation. Specifically, compression screws were associated with higher failure rates, consistent with concerns raised by biomechanical studies regarding their limited rotational stability in certain unstable configurations [35].

In terms of operative parameters, our results showed a shorter operative time and fluoroscopy time for intramedullary nailing, particularly in cases of unstable fractures and when proximal femoral nails were employed. These findings are consistent with prior reviews that noted the technical efficiency and minimally invasive nature of intramedullary nailing, especially in settings requiring rapid stabilization [36,37,38]. Furthermore, the significantly reduced perioperative blood loss associated with nailing observed in this meta-analysis corroborates earlier clinical trials that favored intramedullary constructs for minimizing intraoperative morbidity [39]. The substantial heterogeneity observed in operative time (I^2^ > 90%) and perioperative blood loss (I^2^ = 90.89%) likely reflects variations in several real-world clinical factors across trials. Differences in surgeon experience and familiarity with specific implants can significantly influence operative efficiency and intraoperative blood loss. Additionally, the heterogeneity of implant types (e.g., Ender nails vs. Gamma nails vs. locking plates), as well as differences in surgical technique, fluoroscopic protocols, and intraoperative hemostasis measures, may further account for variability. Institutional perioperative care pathways—such as transfusion thresholds or anesthesia protocols—also vary considerably and could influence intraoperative blood loss reporting. These sources of heterogeneity are inherent in pragmatic surgical trials and should be considered when interpreting the pooled estimates.

Importantly, a notable benefit of intramedullary nailing was a significantly lower risk of deep infection. This may reflect the smaller incisions, shorter operative durations, and less soft tissue disruption inherent to the technique. Similar trends were previously reported by Kuzyk et al., who highlighted infection reduction as one of the key advantages of intramedullary stabilization in trochanteric fractures [40]. However, no differences were found in mortality rates or other systemic complications, underscoring that fixation strategy alone is unlikely to influence broader perioperative risks in this elderly population.

Functional recovery parameters—including hip range of motion and Harris Hip Score—did not significantly differ between groups. This is in line with prior work by Parker et al. [17] and Schipper et al. [35], who found that long-term functional outcomes were more strongly associated with patient-related factors (e.g., age, comorbidity burden) and rehabilitation intensity than fixation method.

From an anatomical standpoint, our findings revealed no difference in shortening, limb length discrepancy, or additional intraoperative fractures. Although previous scoping reviews have debated the potential of plating to better preserve anatomical alignment, our results suggest that both modalities achieve similar biomechanical endpoints when appropriately applied [36,41].

While some have advocated for universal adoption of nailing in all trochanteric fractures, our findings support a more nuanced approach [41]. The data indicate that intramedullary nailing may offer specific perioperative advantages, particularly in unstable fractures, without compromising clinical or functional outcomes. However, fixation method alone does not seem to determine patient-centered endpoints such as mortality or return to baseline function. Therefore, individualized decision making based on fracture configuration, patient physiology, and surgeon expertise remains paramount.

### 4.1. Strengths and Limitations

Our study has several strengths, including the focus on RCTs, the restriction to trochanteric fractures (excluding inter- and subtrochanteric subtypes), and the rigorous subgroup analyses exploring modifiers of treatment effect. However, limitations must be acknowledged. The heterogeneity of implant types, limited blinding in most trials, and absence of long-term outcomes in several studies may introduce bias. Furthermore, the inconsistency in defining and reporting fracture stability highlights the need for standardized classification and reporting practices in future trials [38]. Another important limitation is the lack of patient-reported outcomes (PROs) across the included trials. Key measures such as postoperative pain, patient satisfaction, and quality of life were either inconsistently reported or entirely absent. As these outcomes are increasingly recognized as essential indicators of surgical success—particularly in elderly populations—they should be prioritized in future research to better inform patient-centered decision making.

Notably, only two of the fourteen included RCTs were rated as low risk of bias using the Cochrane RoB 2.0 tool. The most common methodological concerns were related to the lack of blinding—reported in only five studies—and insufficient details on allocation concealment, which were inadequately described in approximately 43% of trials. These limitations are especially relevant for subjective outcomes such as functional scores or operative time, where assessor or surgeon awareness of the intervention may introduce performance or detection bias. While objective outcomes (e.g., mortality, reoperation, implant cut-out) are less susceptible to such bias, the overall internal validity of the meta-analysis may be moderately impacted.

### 4.2. Clinical Implications and Future Directions

The present findings highlight that both intramedullary nailing and plate fixation are effective for managing trochanteric femoral fractures, with no clear superiority in terms of union, reoperation, or mortality. However, the perioperative benefits associated with intramedullary nailing—including reduced operative time, blood loss, and deep infection rates—may offer meaningful advantages in clinical settings where surgical efficiency and morbidity minimization are critical, such as in elderly or polytrauma patients. Surgeons should consider individual fracture morphology, implant availability, and patient comorbidities when selecting the fixation strategy, rather than adhering to a one-size-fits-all approach.

Future research should address the notable gaps identified in this review. Specifically, there is a need for large-scale, multicenter randomized trials with long-term follow-up to better assess functional recovery, quality of life, and implant longevity. Moreover, standardization in fracture classification (e.g., stability grading), outcome reporting, and implant-specific subgroup analyses would enhance the comparability of future studies. Finally, economic evaluations comparing direct and indirect costs of both techniques are warranted to inform cost-effective decision making in healthcare systems increasingly driven by value-based care.

## 5. Conclusions

In conclusion, while both intramedullary nailing and plate fixation remain viable strategies for treating trochanteric femoral fractures, intramedullary nailing appears to confer advantages in operative efficiency, perioperative morbidity, and infection risk. Although intramedullary nailing may confer benefits in terms of operative efficiency, perioperative morbidity, and infection risk, functional outcomes were comparable between the two techniques, underscoring the need for individualized treatment decisions.

## Figures and Tables

**Table 1:** Baseline characteristics of RCTs comparing intramedullary nailing to plate fixation in trochanteric fracture.

Author (YOP)	Design	Country	Stability	Sample		AO/OTA	Nailing	Plating	Gender (M/F)				Age; Mean (SD)				FU (mo)
			Stable/Unstable	Nail	Plate				Nail (M)	Nail (F)	Plate (M)	Plate (F)	Nail		Plate		
Aune (1994) [23]	RCT	Norway	Stable/Unstable	175	203	-	Gamma nail	Compression screw	66	109	89	114	82	49–96	78	45–93	3
Bretherton (2016) [24]	RCT	UK	Unstable	266	272	A1, A2, A3	Intramedullary nail	Sliding hip screw	48	218	54	218	81.3	42–104	80.1	31–103	1.5
Dujardin (2001) [25]	RCT	France	Stable/Unstable	30	30	-	Mini-invasive static nail	Dynamic hip screw	6	24	6	24	83	9.4	84	6.2	6
Karn (2011) [26]	RCT	Nepal	Stable/Unstable	30	30	-	Proximal femoral nail	Sliding hip screw	18	12	12	18	66.56	53–100	67.8	50–87	6
Kassem (2022) [27]	RCT	Egypt	Unstable	34	34	A2	Proximal femoral nail	Dynamic hip screw	-	-	-	-	70.8	7.7	68.7	8.7	12
Parker (2012) [28]	RCT	UK	Stable/Unstable	300	300	B2.1, A1, A2, A3	Targon Proximal femoral nail	Sliding hip screw	52	248	69	231	82.4	26–104	81.4	27–104	12
Parker (2017a) [18]	RCT	UK	Stable/Unstable	500	500	A1, A2, A3	Intramedullary nail	Sliding hip screw	112	388	116	384	82.2	26–104	82.1	25–105	2
Parker (2017b) [19]	RCT	UK	Stable/Unstable	200	200	A1, A2, A3	Targon Proximal femoral nail	Sliding hip screw	60	140	47	153	82	36–101	83.2	25–105	12
Schemitsch (2023) [29]	RCT	12 countries	Stable/Unstable	418	415	A1, A2	Intramedullary nail	Sliding hip screw	153	265	138	277	78.2	26–102	78.8	18–100	12
Singh (2017) [30]	RCT	India	Unstable	23	22	A2, A3	Proximal femoral nail	Locking compression plate	9	14	7	15	58.3	9.3	60.5	8.1	24
Stark (1992) [31]	RCT	Sweden	Stable/Unstable	36	56	-	Ender nails	Sliding hip screw	12	24	17	39	74	-	75	-	6
Utrilla (2005) [32]	RCT	Spain	Stable/Unstable	104	106	-	Gamma nail	Compression hip screw	38	66	78	28	80.6	7.5	79.8	7.3	12
Zehir (2015) [33]	RCT	Turkey	Unstable	96	102	A2	Proximal femoral nail anti-rotation	Dynamic hip screw	37	59	39	63	77.22	6.82	76.86	6.74	6
Zou (2009) [34]	RCT	China	Stable/Unstable	58	63	A1, A2, A3	Proximal femoral nail anti-rotation	Dynamic hip screw	-	-	-	-	-	-	-	-	12

YOP: year of publication; RCT: randomized controlled trial; M: male; F: female; SD: standard deviation; FU: follow-up; mo: month.

**Table 2:** A summary of the meta-analytic estimates for the risk of complications between intramedullary nailing and plate fixation in trochanteric fractures.

Complication	Studies	OR	95% CI	I^2^ (%)	*p*-Value for I^2^
AVN	3	0.49	0.08–2.94	0%	0.37
DVT	4	1.18	0.64–2.19	0%	1.00
Deep infection	8	0.24	0.08–0.73	0%	1.00
Heart failure	2	0.54	0.15–1.98	24.04%	0.25
Hematoma	3	0.44	0.11–1.74	0%	0.95
Infection (not classified)	2	0.58	0.13–2.60	0%	0.65
Non-union	4	1.44	0.39–5.35	0%	0.9
Pulmonary embolism	2	1.36	0.30–6.14	0%	0.84
Pneumonia	2	1.32	0.69–2.52	0%	0.74
Pressure ulcers	2	1.03	0.55–1.92	0%	0.83
Superficial infection	7	0.92	0.53–1.62	0%	0.95

OR: odds ratio; CI: confidence interval; I^2^: a measure of heterogeneity (significant if >50%).

## Data Availability

The original contributions presented in this study are included in the article/Supplementary Materials. Further inquiries can be directed to the corresponding author.

## Supplementary Materials

The following supporting information can be downloaded at: https://www.mdpi.com/article/10.3390/jcm14155492/s1. Figure S1: Forest plot showing the risk of reoperation in intramedullary nailing versus plate fixation in trochanteric fracture; Figure S2: Leave-one-out sensitivity analysis of the reoperation rate outcome; Figure S3: Forest plot showing the difference in union time between intramedullary nailing and plate fixation in trochanteric fracture; Figure S4: Forest plot showing the risk of implant cut-out in intramedullary nailing versus plate fixation in trochanteric fracture; Figure S5: Leave-one-out sensitivity analysis of operative time; Figure S6: Forest plot showing the difference in perioperative blood loss between intramedullary nailing and plate fixation in trochanteric fracture; Figure S7: Forest plot showing the risk of blood transfusion in intramedullary nailing versus plate fixation in trochanteric fracture; Figure S8: Forest plot showing the difference in length of hospital stay between intramedullary nailing and plate fixation in trochanteric fracture; Figure S9: Forest plot showing the risk of additional fractures in intramedullary nailing versus plate fixation in trochanteric fracture; Figure S10: Forest plot showing the risk of additional fractures in intramedullary nailing versus plate fixation in trochanteric fracture, stratified by nail/plate type, age, and fracture stability; Table S1: The detailed search syntax employed in the database search of this systematic review.

## Abbreviations

The following abbreviations are used in this manuscript:

AVN	Avascular necrosis
CI	Confidence interval
DVT	Deep vein thrombosis
FU	Follow-up
IMN	Intramedullary nailing
I^2^	Measure of heterogeneity
LD	Linear dichroism
MD	Mean difference
MDPI	Multidisciplinary Digital Publishing Institute
OR	Odds ratio
PF	Plate fixation
PRISMA	Preferred Reporting Items for Systematic Reviews and Meta-Analyses
RCT	Randomized controlled trial
RoB 2.0	Revised Cochrane Risk of Bias tool for Randomized Trials
ROM	Range of motion
SD	Standard deviation
TLA	Three-letter acronym
