# Supplementary material for: Intramedullary Nailing vs. Plate Fixation for Trochanteric Femoral Fractures: A Systematic Review and Meta-Analysis of Randomized Trials
Source: J Clin Med. 2025 Aug 4;14(15):5492. doi: 10.3390/jcm14155492 (PMC12347882; doi:10.3390/jcm14155492)
Supplement: Supplementary file 1 [file jcm-14-05492-s001.zip › Table S1.docx]

**Table S1.** The detailed search syntax employed in the database search of this systematic review

| Database | No. | Search Syntax | Results |
| --- | --- | --- | --- |
| PubMed | #1 | Femur[tiab] OR femoral[tiab] OR "Femur"[Mesh] OR Hip[tiab] OR "Hip"[Mesh] | 345552 |
|  | #2 | Fracture*[tiab] | 326159 |
|  | #4 | Nail*[tiab] | 41369 |
|  | #5 | Plat*[tiab] | 880928 |
|  | #6 | Random*[tiab] OR "Randomized Controlled Trial" [Publication Type] | 1651168 |
|  | #7 | #1 AND #2 AND #3 AND #4 AND #5 AND #6 | 99 |
| Scopus | #1 | TITLE-ABS-KEY (Femur) OR TITLE-ABS-KEY (femoral) OR TITLE-ABS-KEY (Hip) | 535308 |
|  | #2 | TITLE-ABS-KEY (Fracture*) | 1014828 |
|  | #4 | TITLE-ABS-KEY (Nail*) | 86657 |
|  | #5 | TITLE-ABS-KEY (Plat*) | 3065795 |
|  | #6 | TITLE-ABS-KEY (Random*) | 3322770 |
|  | #7 | #1 AND #2 AND #3 AND #4 AND #5 AND #6 | 261 |
| Web of Science | #1 | AB=Femur OR AB=femoral OR AB=Hip | 285662 |
|  | #2 | AB=Fracture* | 484953 |
|  | #4 | AB=Nail* | 33641 |
|  | #5 | AB=Plat* | 1842120 |
|  | #6 | AB=Random* | 2187348 |
|  | #7 | #1 AND #2 AND #3 AND #4 AND #5 AND #6 | 118 |
| Cochrane Registry | #1 | Femur OR femoral OR Hip | 45327 |
|  | #2 | Fracture* | 31086 |
|  | #4 | Nail* | 4548 |
|  | #5 | Plat* | 79458 |
|  | #6 | Random* | 1422676 |
|  | #7 | #1 AND #2 AND #3 AND #4 AND #5 AND #6 | 161 |
| Clinicaltrials.gov | Condition/disease | Femoral Fractures | - |
|  | Other terms | Plate | - |
|  | Intervention/treatment | Nail | - |
|  | Total | Filter applied: Completed | 10 |
| Google Scholar | With all of the words | random fracture nail plate | - |
|  | With the exact phrase | - | - |
|  | With at least one of the words | Femur hip | - |
|  | Total | As per recent recommendations, only the first 200 records were selected | 200 |
