# Supplementary figures and images for: Intramedullary Nailing vs. Plate Fixation for Trochanteric Femoral Fractures: A Systematic Review and Meta-Analysis of Randomized Trials
Source: J Clin Med. 2025 Aug 4;14(15):5492. doi: 10.3390/jcm14155492 (PMC12347882; doi:10.3390/jcm14155492)

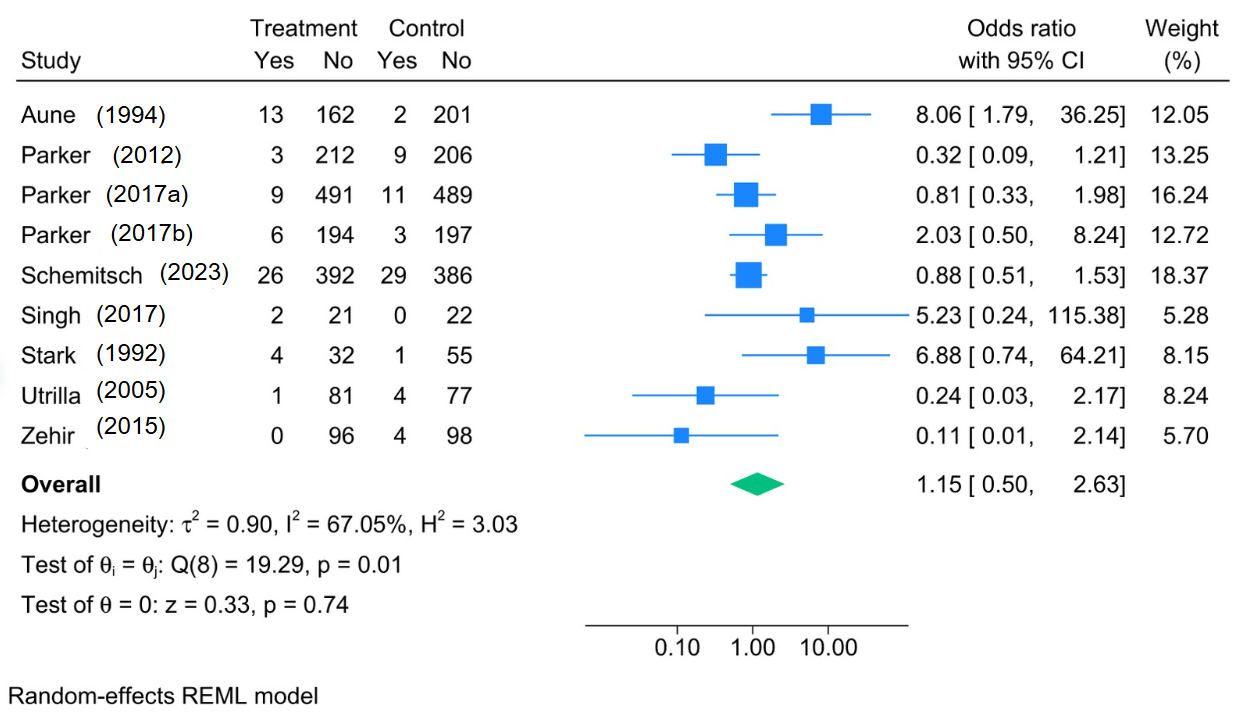

Supplement: Supplementary file 1 [file jcm-14-05492-s001.zip › Figure S1.tif]

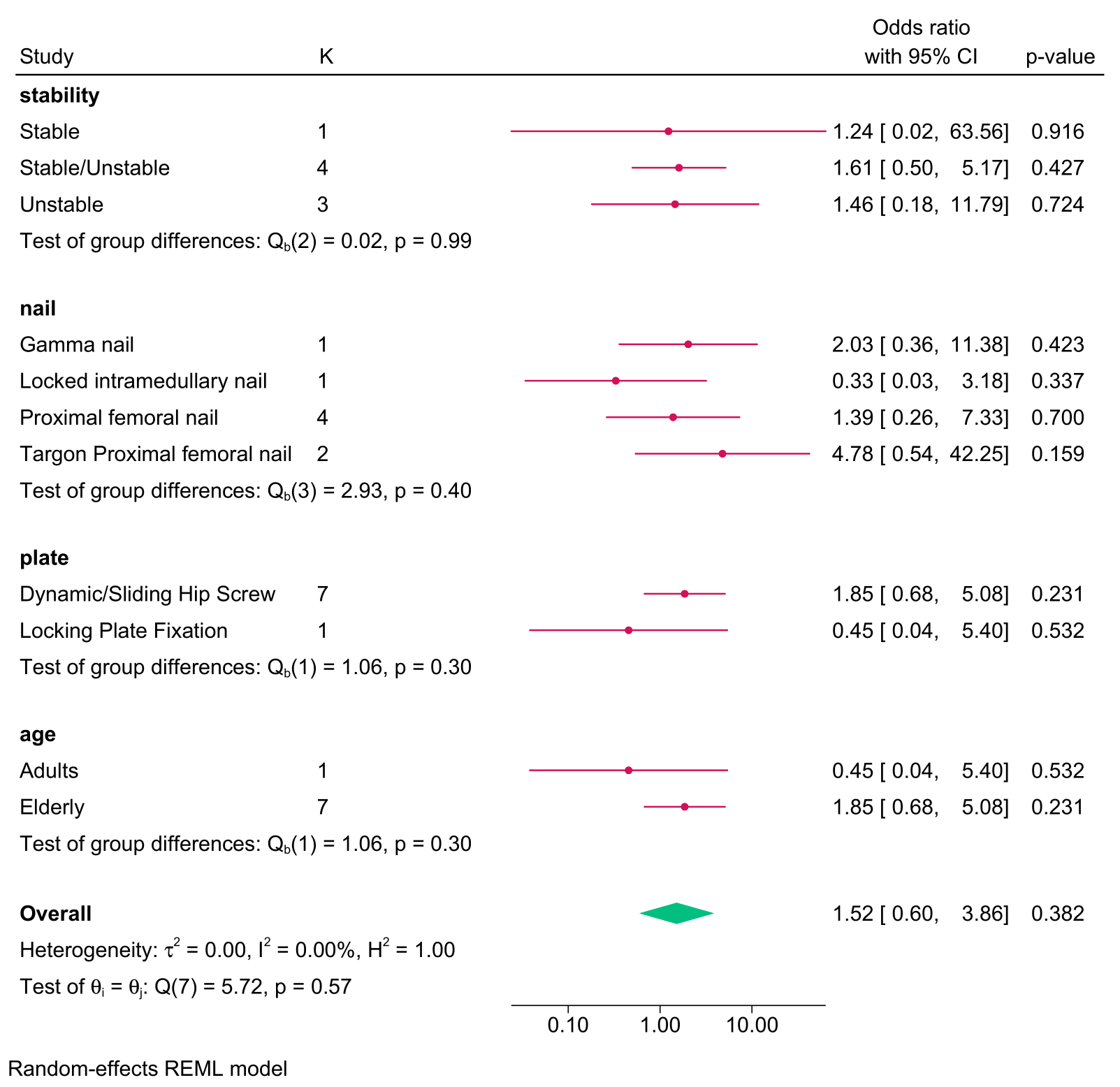

Supplement: Supplementary file 1 [file jcm-14-05492-s001.zip › Figure S10.tiff]

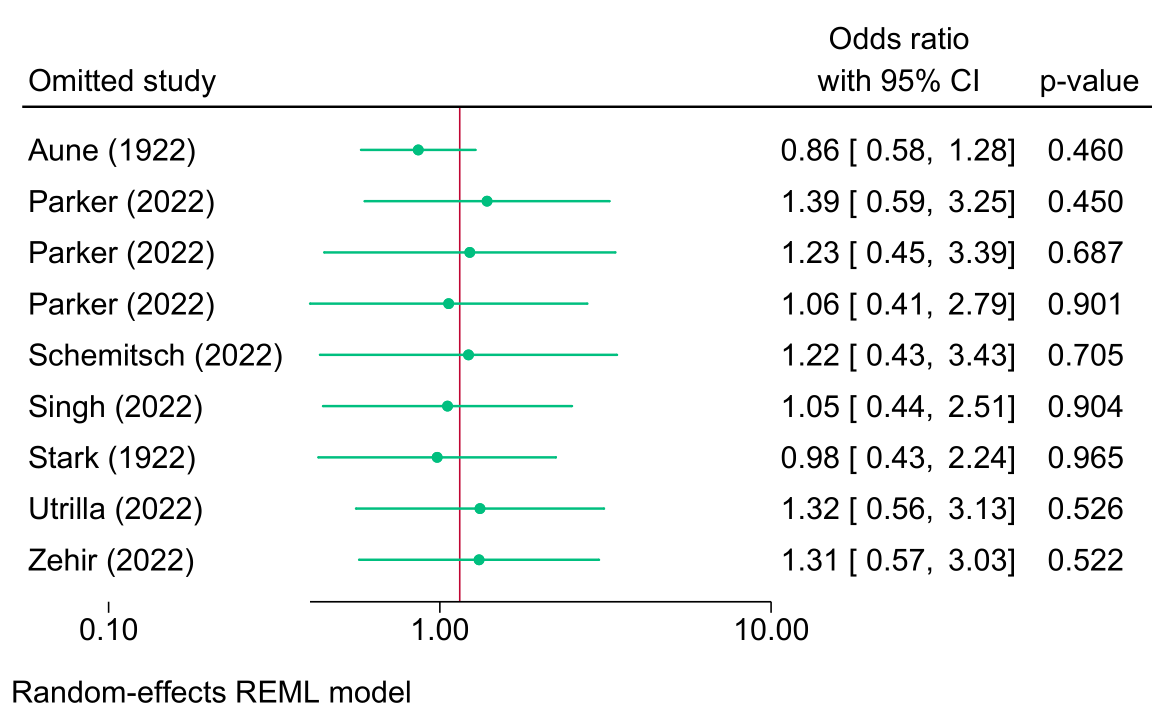

Supplement: Supplementary file 1 [file jcm-14-05492-s001.zip › Figure S2.tiff]

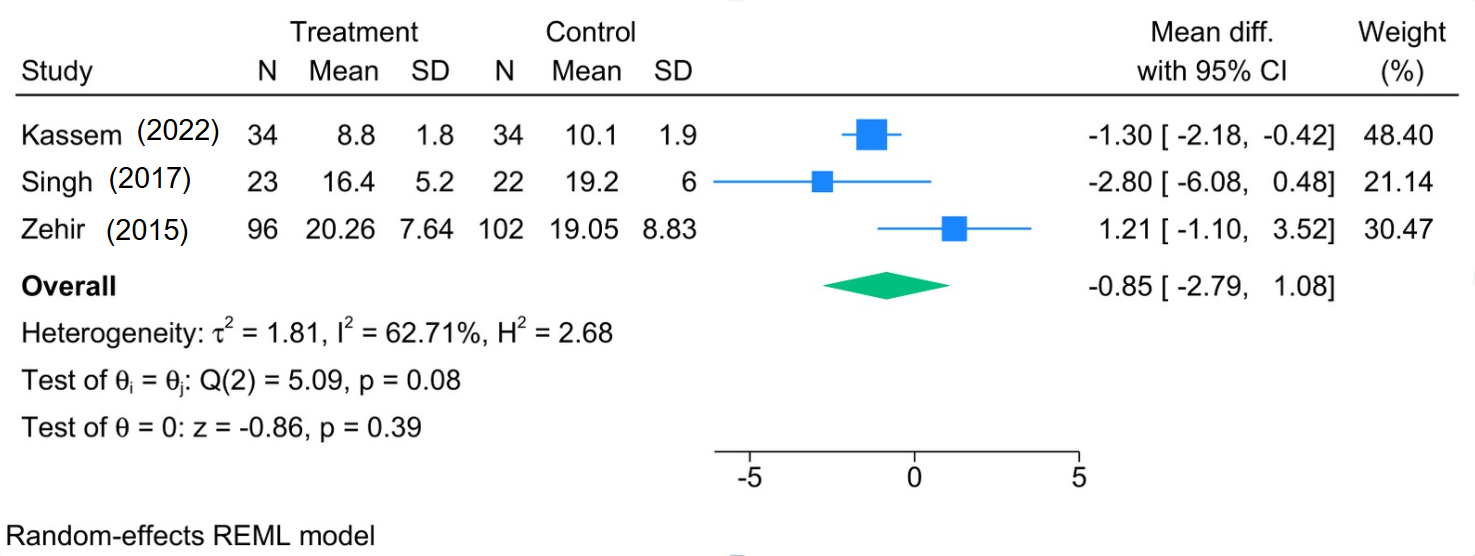

Supplement: Supplementary file 1 [file jcm-14-05492-s001.zip › Figure S3.tif]

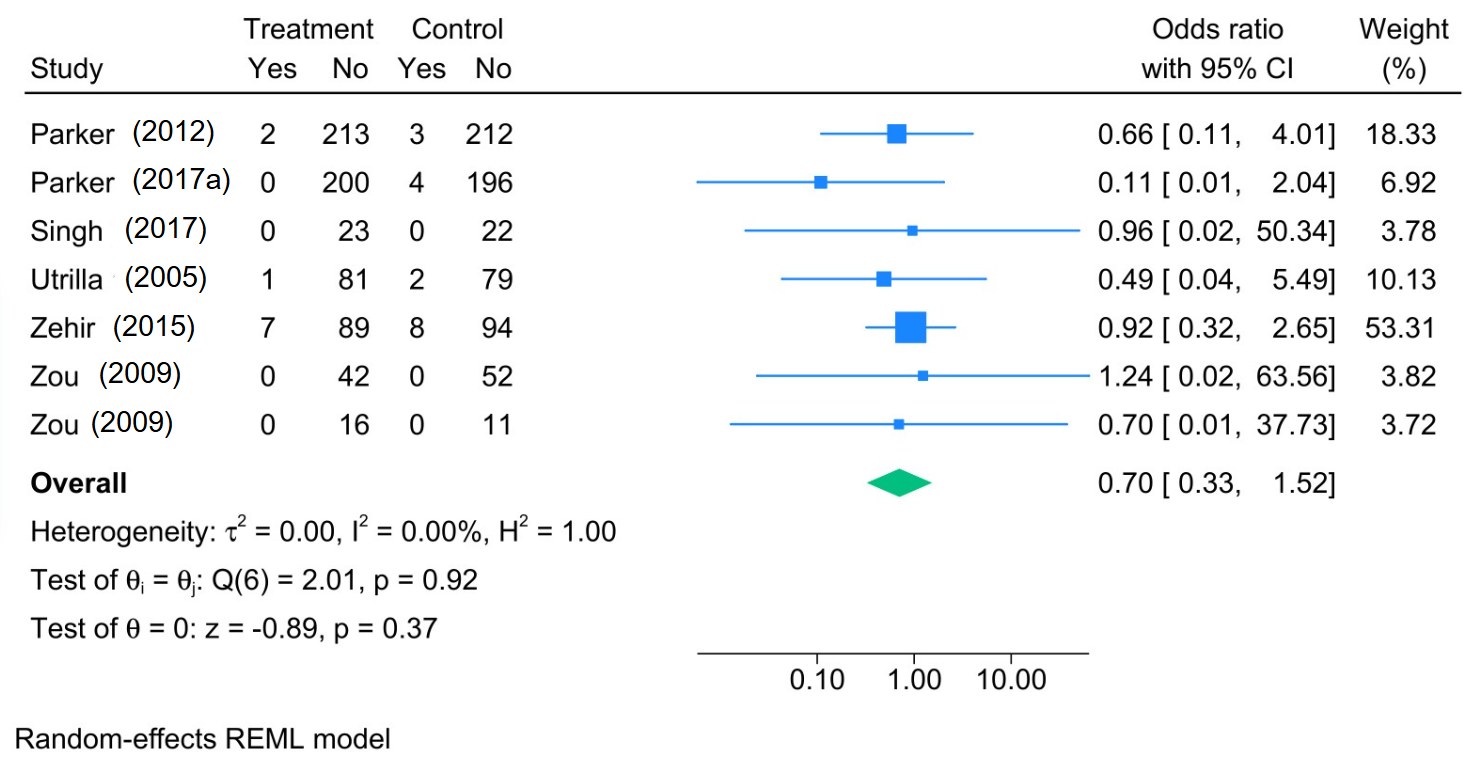

Supplement: Supplementary file 1 [file jcm-14-05492-s001.zip › Figure S4.tif]

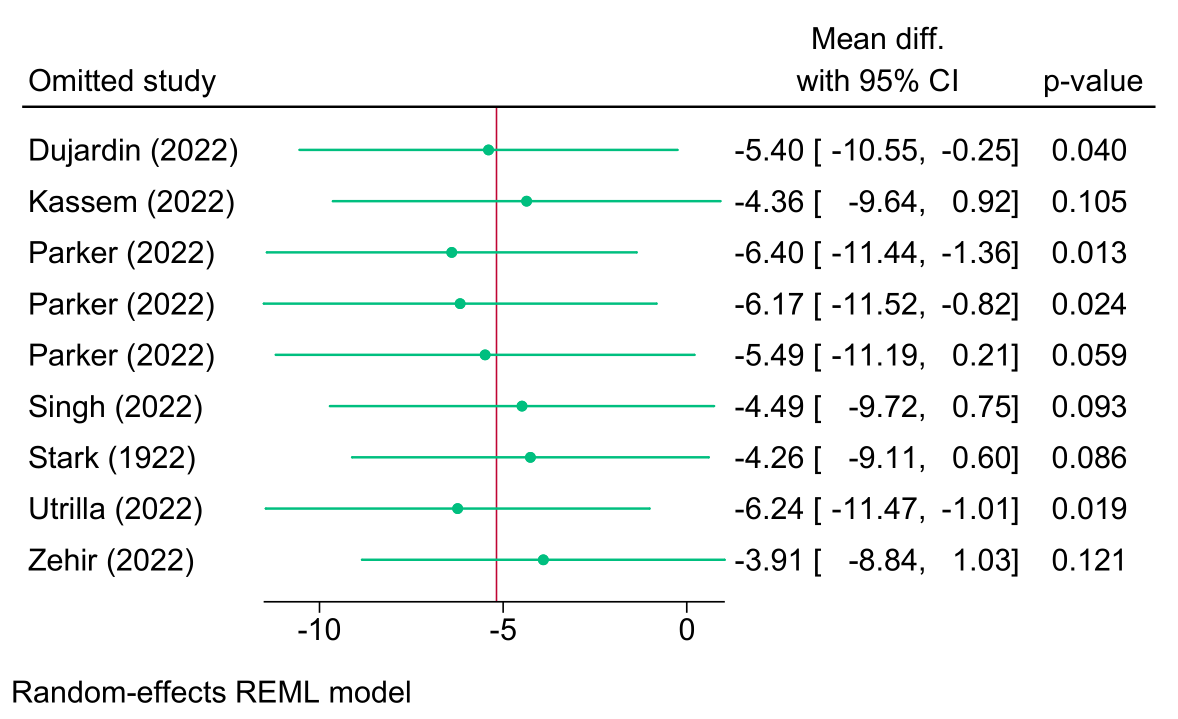

Supplement: Supplementary file 1 [file jcm-14-05492-s001.zip › Figure S5.tiff]

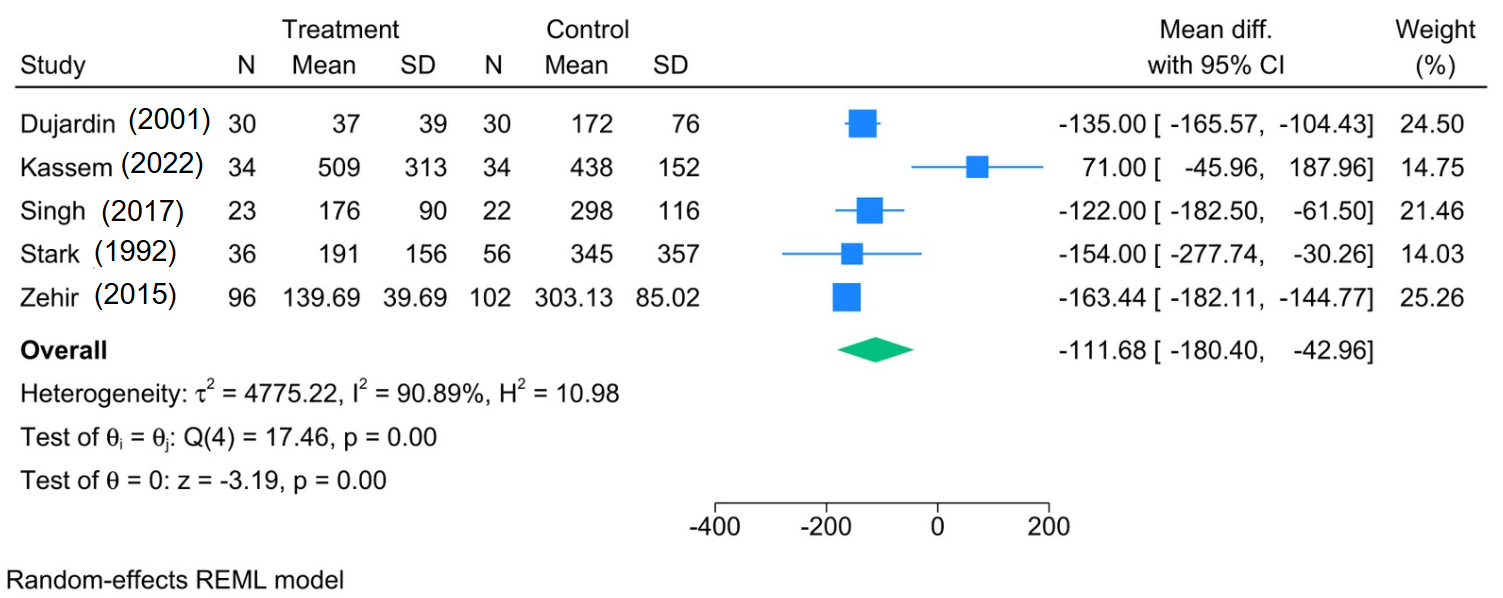

Supplement: Supplementary file 1 [file jcm-14-05492-s001.zip › Figure S6.tif]

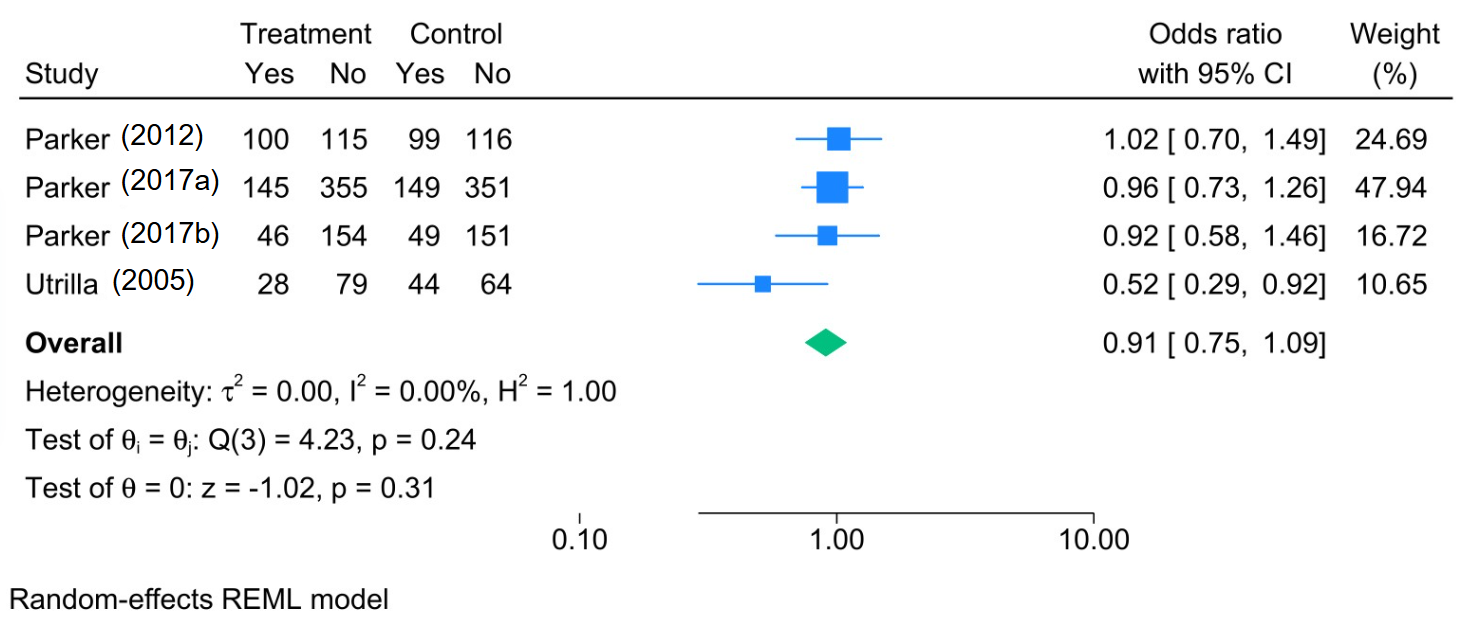

Supplement: Supplementary file 1 [file jcm-14-05492-s001.zip › Figure S7.tif]

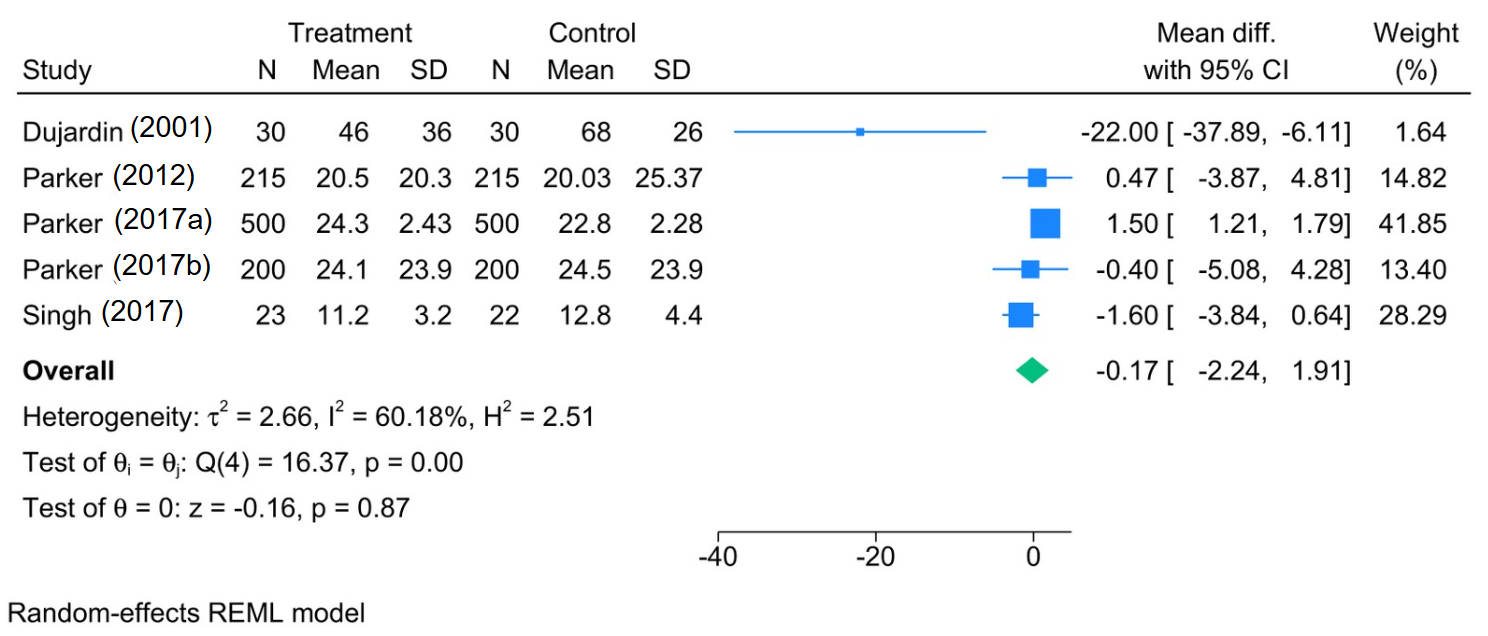

Supplement: Supplementary file 1 [file jcm-14-05492-s001.zip › Figure S8.tif]

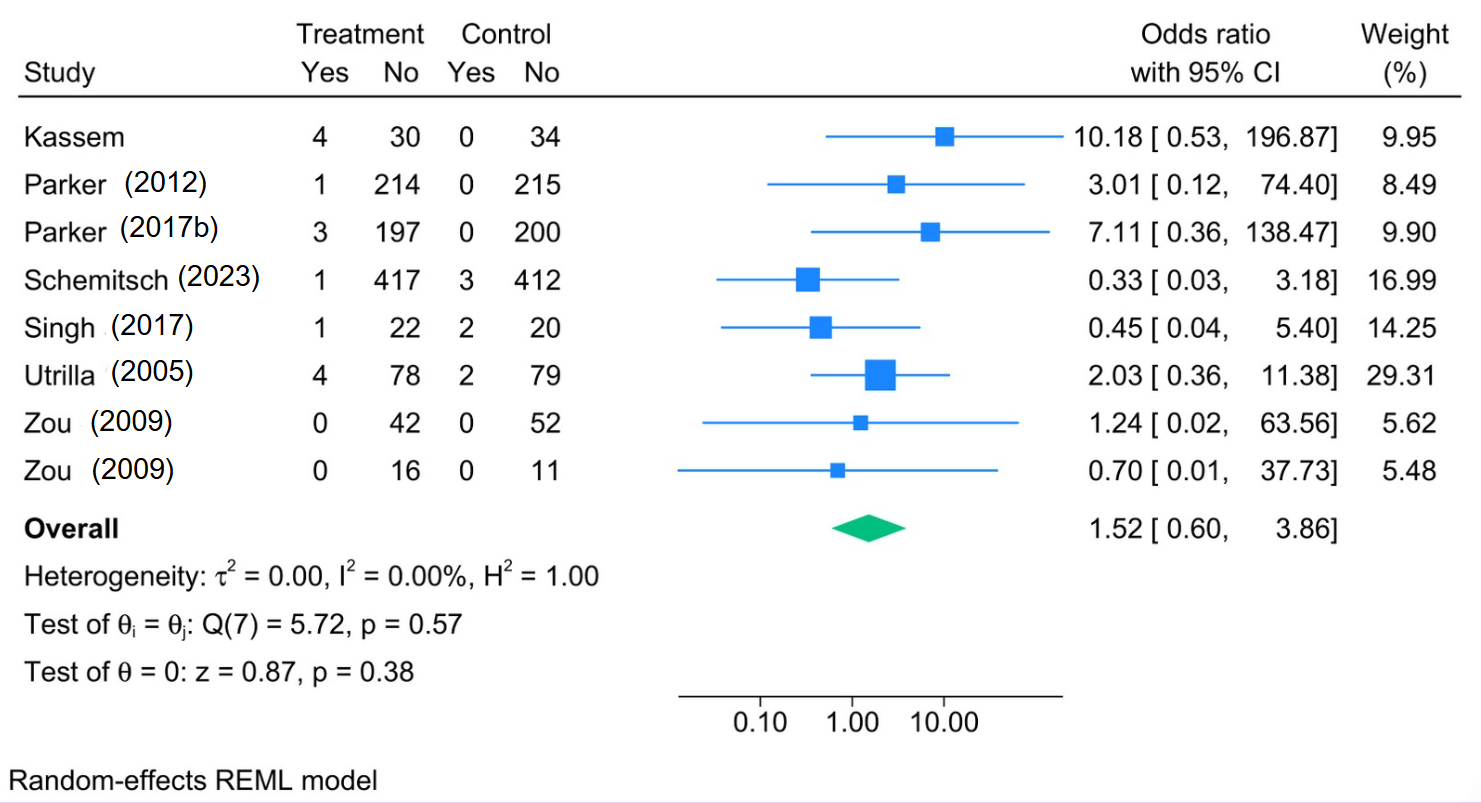

Supplement: Supplementary file 1 [file jcm-14-05492-s001.zip › Figure S9.tif]
